# Investigation of Camphor Effects on *Fusarium graminearum* and *F. culmorum* at Different Molecular Levels

**DOI:** 10.3390/pathogens7040090

**Published:** 2018-11-22

**Authors:** Aylin Gazdağlı, Özlem Sefer, Emre Yörük, Gülin İnci Varol, Tuğba Teker, Gülruh Albayrak

**Affiliations:** 1 Institute of Graduate Studies in Sciences, Programme of Molecular Biology and Genetics, Istanbul University, Suleymaniye, Istanbul 34116, Turkey; aylin.gazdagli@gmail.com (A.G.); varol.gulin@gmail.com (G.İ.V.); tugba.teker@ogr.iu.edu.tr (T.T.); 2 Faculty of Arts and Sciences, Department of Molecular Biology and Genetics, Istanbul Yeni Yuzyil University, Cevizlibag, Istanbul 34010, Turkey; ozlemsefer@outlook.com; 3 Faculty of Sciences, Department of Molecular Biology and Genetics, Istanbul University, Vezneciler, Istanbul 34134, Turkey; gulruh@istanbul.edu.tr

**Keywords:** apoptosis, camphor, epigenetics, gene expression, oxidative stress

## Abstract

*Fusarium graminearum* and *F. culmorum* are phytopathogens, which cause destructive diseases in cereals. Epidemics of these phytopathogens are caused by mycotoxin contamination and the reduction of crop quality. In this study, the alteration due to in vitro camphor treatment on *F. culmorum* 9F and *F. graminearum* H11 isolates was investigated in terms of epigenetic, cellular, and transcription levels. Camphor with different concentrations (0.2, 0.4, 0.8, 1, 2, and 4 µg/µL) was applied to potato dextrose agar (PDA) growth media. The minimum inhibitory concentration (MIC) and the half maximal inhibitory concentration (IC_50_) were calculated as 2 and 1 µg/µL, respectively. *hog1*, *mst20*, *CAT*, *POD*, *mgv1*, *stuA*, and *tri5* genes, which are related to various cellular processes and pathogenesis, were examined by qPCR assay. qPCR analysis showed that camphor treatment leads to the downregulation of *tri5* expression but the upregulation of the remaining genes. Apoptosis and oxidative stress were confirmed via acridine orange/ethidium bromide (AO/EB) and dichlorofluorescin diacetate (DCF-DA) staining, respectively. Moreover, coupled restriction enzyme digestion-random amplification (CRED-RA) assay, used for DNA methylation analysis, was carried out to evaluate epigenetic alterations. The decrease in genomic template stability (GTS) values, which resulted due to the alterations in random amplified polymorphic DNA (RAPD) profiles caused by camphor treatment, were detected as 97.60% in *F. culmorum* 9F and 66.27% in *F. graminearum* H-11. The outer and inner methylated cytosine profiles are determined by CRED-RA assay as type I–IV epigenetic alterations. The outcomes indicated that camphor could lead to alterations at several molecular levels of *F. graminearum* and *F. culmorum*.

## 1. Introduction

*F. graminearum* and *F. culmorum* are species of the phytopathogenic fungi genus, *Fusarium*. Both species have been reported as primary causal agents of various plant diseases worldwide. In particular, *F. graminearum* is determined as the major causal agent of *Fusarium* head blight (FHB) and crown rot, particularly in China and the USA [1,2,3,4,5]. They are not only pathogens responsible for the destructive effects on crops and for the economic losses, but they also produce several mycotoxins, such as deoxynivalenol (DON) and zearalenone (ZEN), which are hazardous to humans and other animals [1,6].

*F. graminearum* is a hemi-biotrophic fungus with asexual (*F. graminearum*, anamorph) and sexual (*Gibberella zeae*, telemorph) stages. The alleles required for sexual reproduction, which are located in the genome, are *MAT1-1* and *MAT1-2* [7,8]. The genome size (*n* = 4) of the *F. graminarum* PH-1 strain, which is the reference genome, is 36.1 Mb. The PH-1 strain genome carries approximately 14,000 genes and is responsible for the synthesis of approximately 180,000 proteins. Further information on annotated genes, related to the transcription process, membrane transport system, and aggressiveness, can been accessed in GenBank [9].

*F. culmorum*, in comparison to *F. graminearum*, is a monophyletic species with an unknown sexual stage. However, in recent years, parasexual reproduction has been investigated for *F. culmorum* [1,10]. The pathogen has a wide host range, and this necrotrophic fungus is a secondary phytopathogen of FHB and crown rot. Currently, a complete annotated genome is not present in databases for *F. culmorum*, though partial annotations for chromosomes of the FcUK99 strain can be accessed on GenBank.

In the fight against these *Fusarium* species, it is crucial to develop disease-resistant plant cultivars and to use antagonistic microorganisms. However, these costly and laborious strategies require extended periods of time, as well as advanced laboratory skills and experience [11,12,13]. Furthermore, up-to-date, commonly used antifungal agents such as thiophanate methyl, carbendazim, and propiconazole are preferred against *Fusarium* species, but these species have developed resistance against such common antifungals [14,15,16]. So, many novel chemicals must be tested in terms of their antifungal effects. Therefore, an in vitro demonstration of fungicidal effects of essential oils obtained from plants could lead to in planta fungicide treatments as disease management.

The major components of essential oils obtained from plant extracts (kapyrrole derivatives, indole derivatives, etc.) have been used to inhibit fungal growth [17,18,19]. One of the major components of essential oils, camphor (C_10_H_16_O), is a bicyclic monoterpene ketone widely found in several aromatic plants such as *Cinnamonum camphora*, *Piper capense*, and *Salvia officinalis*. Even though large doses of camphor show toxicity for humans, it has been used in traditional medicine [20] and different commercial cosmetic products with limited doses. As far as we know, there is no study on the potential antifungal effects of camphor in *F. graminearum* and *F. culmorum.* In this study, camphor’s effects on *F. graminearum* and *F. culmorum* in terms of the in vitro growth capacity of fungal cells, alterations of specific gene expression levels, apoptosis, oxidative stress, and camphor’s epigenetic alteration capacity on fungal cells were investigated for the first time.

## 2. Results

### 2.1. Antifungal Application Analysis

*Fusarium* species did not show growth on potato dextrose agar (PDA) +2 µg/µL camphor. Thus, the minimum inhibitory concentration (MIC) was determined as 2 µg/µL by common protocol for agar dilution techniques, as a reported by Irzykowska [21]. Then, the half maximal inhibitory concentration (IC_50_) was calculated as 1 µg/µL. This concentration was used for experiments in further studies.

### 2.2. Gene Expression Analysis

**Figure 1:**
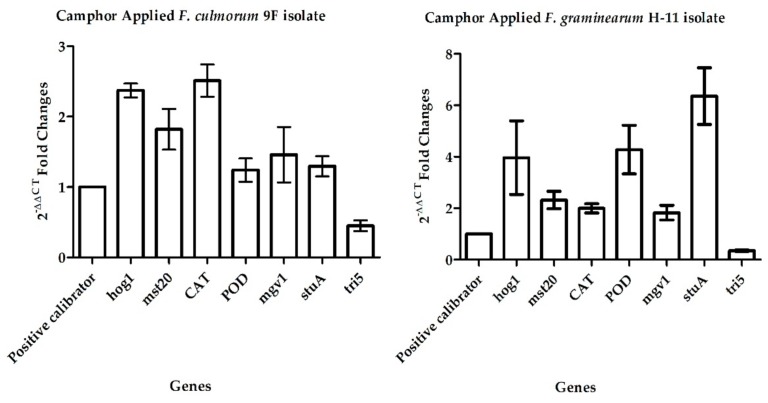
Fold changes in gene expressions in response to camphor.

cDNAs were converted from high-quality (∆_260/280_= 1.9–2.0) and a high amount (0.5–2 μg μL^−1^) of total RNA molecules. *hog1*, *mst20*, *CAT*, *POD*, *mgv1*, *stuA*, and *tri5* gene expression levels were determined. In quantitative polymerase chain reaction (qPCR) analysis, the mean E (efficiency) value, which was calculated using Cp (crossing point) values related to log series of cDNAs, was obtained as 2.131 ± 0.059, and the mean melting score was 0.89 ± 0.007. In this study, both types of data showed that qPCR was applied very efficiently. The 2^−ΔΔCT^ values (1.241 ± 0.16 to 6.359 ± 1.01) for experimental sets of target genes are given in Table 1. After normalization, *hog1* and *mst20* (associated with apoptosis), *CAT* and *POD* (responsible for oxidative stress), as well as *mgv1* and *stuA* (liable for cell survival and viability) expressions increased, ranging from 1.826 ± 0.28 to 6.359 ± 1.01 by camphor treatment. This revealed that these genes were upregulated. The decrease of *tri5* (responsible for mycotoxin production) expressions (from 0.349 ± 0.02 to 0.449 ± 0.07) was also detected (Figure 1). These results were found to be significantly different in statistical analysis (*p* < 0.05).

### 2.3. Staining Analysis

The presence of late-phase apoptotic cells was demonstrated by AO/EB (acridine orange/ethidium bromide) staining. Healthy cells in control groups radiated green fluorescence dye in both *Fusarium* species. In camphor-treated experimental sets, light yellow- to light brown-colored mycelia showed the occurrence of a process similar to apoptosis. The fluorescence density of EB was greater than that in the experimental sets.

**Figure 2:**
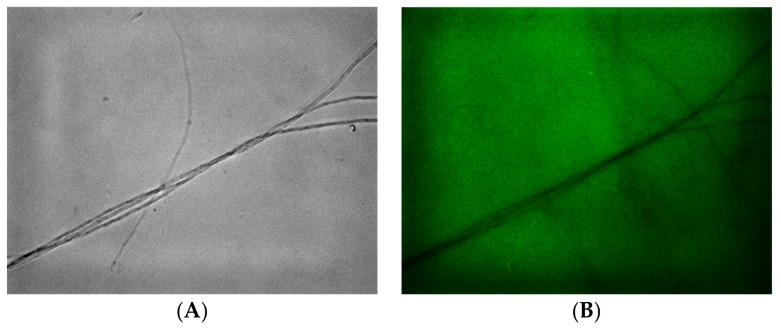

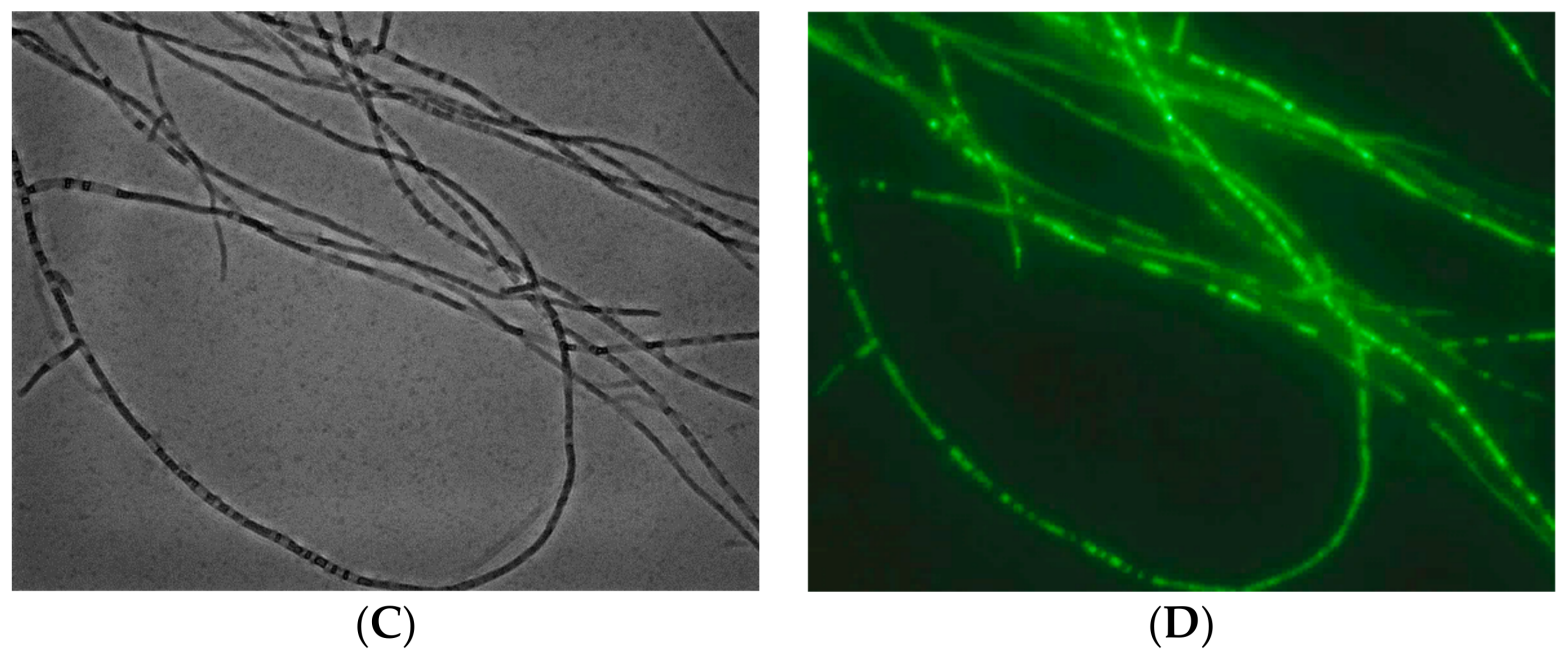
(**B**,**D**) Fluorescein isothiocyanate (FITC) and (**A**,**C**) daylight filter-captured profile of cells treated with camphor (**C**,**D**) and control sets (**A**,**B**).

The presence of ROS (reactive oxygen species) activity was investigated in experimental sets with green luminescence, using DCF-DA (2′,7′-dichlorodihydrofluorescein diacetate) dye. No colored cells were observed in the control groups (Figure 2). Mycelial growth was identified in camphor-treated cells at the days 5, 7, and 14 of incubation.

### 2.4. Antioxidant Activities and Thin-Layer Chromatography (TLC)

Abiotic stress was confirmed by both spectrophotometrical analysis and qPCR. *CAT* and *POD* gene expression levels were high in both camphor-treated *F. graminearum* (H-11) and *F. culmorum* (9F) isolates. Upregulated *CAT* and *POD* gene expression levels and enzyme activity values (significant differences; *p* < 0.05) showed that camphor caused oxidative stress. CAT (catalase) activities were calculated as 1.73 ± 0.6 (*p* < 0.05) and 4.24 ± 2.01 (*p* < 0.01) for *F. culmorum* 9F and *F. graminearum* H-11 isolates, respectively. Similarly, POD (peroxidase) activities for *F. culmorum* 9F and *F. graminearum* H-11 isolates were recorded as 1.5 ± 0.5 (*p* < 0.05) and 1.5 ± 0.5 (*p* < 0.05), respectively. At the end of the TLC analysis, in control sets, spots corresponding to the DON mycotoxin with an Rf (retention factor) value of 0.28 were detected. In the experimental sets, the spots appeared to be insignificant for certain samples and were not observed for other samples. Therefore, the data were obtained as a result of the inhibition of DON production by camphor.

### 2.5. Coupled Digestion-Random Amplification (CRED-RA) and Random Amplified Polymorphic DNA (RAPD) Analyses

A total of 15 primers were used for RAPD and CRED-RA analyses. No amplification product was obtained from the five oligonucleotide primers (OPA-04, OPA-05, OPA-06, OPC-04, OPC-05). However, the polymerase chain reaction (PCR) procedure worked properly for the remaining 10 oligonucleotide primers and PCR fragments were obtained in 12 experimental sets (Table 2).

Genomic template stability (GTS) analysis was carried out with 86 bands obtained from non-digested sets of IC_50_ and from the control groups. Minimum amplification products (5) were obtained from OPB-07, while maximum fragments (13) were detected from OPA-01. The GTS values for IC_50_ groups of *F. culmorum* 9F and *F. graminearum* H-11 were detected as 97.60% and 66.27%, respectively. This analysis showed that camphor treatment with increasing concentrations caused a decrease in genomic stability.

Due to camphor treatment, type I–IV epigenetic alterations, which represent the outer and inner methylated cytosine profiles, were detected in this analysis. The epigenetic alterations related to *HapII* and *MspI* digestion profiles of the experimental groups are shown in Table 3.

The average polymorphisms for epigenetic alterations ranged from 2.85% to 55.7% for *HapII* and *MspI* analyses. Polymorphic changes based on epigenetic alterations were characterized by the band intensity and the loss or addition of a band.

## 3. Discussion

Isolates with high genotypic and chemotypic diversity were obtained from diseased plants cultivated in agro-ecological regions with different climatic conditions. Class B trichothecene chemotypes and molecular marker-based genotypic diversity have been studied most frequently. Chemotypes with 15-ADON and 3-ADON have been reported to be the predominating chemotypes for *F. graminearum* and *F. culmorum*, respectively [1,2,3]. Additionally, the regulation of mycotoxin biosynthesis in vitro and in planta has also been well studied in *Fusarium* spp. [22,23,24]. Investigations related to the discovery of novel compounds can lead to the discovery of potential fungicides or chemicals with antifungal effects. These could be useful for the development of both local and global disease management strategies.

Fungicide treatment in *F. graminearum*- and *F. culmorum*-infected fields presents a promising strategy for dealing with FHB and crown rot epidemics. However, resistance developing by both *Fusairum* species against the common fungicidal compounds, which are used in crops, is an observed difficulty. Genetic diversity becomes effective for the development of resistance to these chemicals [14,15,16,25]. Currently, there is limited knowledge about the epigenetic or biochemical background of this resistance. In this study, the antifungal effects of camphor—one of the compounds of plant essential oils—were investigated by molecular approaches.

Camphor can be a promising agent for in vitro and/or in planta disease management, because it is less harmful to human and animal health. So far, the antifungal potential of some chemical compounds derived from plant or bacteria have been investigated in *Fusarium* species. However, comprehensive data were not obtained from those studies based on the investigation of the macroconidium reproduction capacity, radial growth rate, and inhibition of mycotoxin biosynthesis alterations [17,18,25,26]. Gene alterations, which are related to important processes in the fungal life cycle, the detection of apoptosis-like processes, and the determination of the presence of oxidative stress, have not been investigated in *F. graminearum* and *F. culmorum*. In this study, camphor’s potential antifungal effects were investigated in *F. graminearum* and *F. culmorum* for the first time in terms of gene expression, epigenetics, and cellular analysis.

In this study, the MIC values for these two species were found at moderate levels as compared with some other plant essential oils such as eugenol, vanillin, dimethyl pyrrole, etc. In addition, a relatively higher level of mycotoxin inhibition was also detected via qPCR [17,18,26,27,28,29,30]. IC_50_ values of 1 µg/µL were used in further studies.

Alterations in *hog1*, *mst20*, *CAT*, *POD*, *mgv1*, *stuA*, and *tri5* expressions were evaluated in this study for the first time for the antifungal effects of camphor in terms of molecular genetic analysis. *hog1* (MAP kinase) and *mst20* (serine/threonine-protein kinase) genes are associated with apoptosis-like processes. The expression of *CAT* (catalase) and *POD* (peroxidase) genes is regulated as a response to oxidative stress. *mgv1* (mitogen-activated protein kinase gene) and *stuA* (apses transcriptional factor gene) are essential genes required for cell viability and survival. *tri5* (trichodiene synthase gene) encodes the enzyme which is responsible for the first reaction of the *tri5* gene cluster.

*mgv1* and *stuA*, which can be accepted as positive calibrator genes for changes in homeostasis of the fungal life cycle, showed upregulation as a response to camphor treatment. These data are important since they represent the potential for physiological alterations in camphor-treated *Fusarium* isolates. The presence of an apoptosis-like process was detected by gene expression analysis and fluorescence analysis. In addition, antioxidant activity by spectrophotometer assay was used to show the presence of oxidative stress. Findings showed that camphor can be a potential agent for *F. graminearum* and *F. culmorum* by triggering an apoptosis-like process and oxidative stress. Additionally, *tri5* downregulation, which was demonstrated by qPCR and TLC assays, signified that *F. graminearum* and *F. culmorum* might exhibit reduced DON-producer phytopathogens via camphor treatment. In RAPD and CRED-RA analyses, both genomic template stability and epigenetic alterations were detected. A moderate level of alterations in genomic template stability and type I/IV and type II/III methylation profiles were obtained in this study [19,30]. However, alterations on chromosomal regions would increase the effects of camphor on the aggressiveness and viability of *F. graminearum* and *F. culmorum*. In general, it seems that camphor can be used as a useful agent with potential antifungal effects against *Fusarium* spp.

## 4. Materials and Methods

### 4.1. Antifungal Treatment

H-11 *F. graminearum* isolate was obtained from diseased corn samples in Korea by Dr. Therese Lee and 9F *F. culmorum* isolate was obtained from diseased wheat spike in Turkey by Dr. Berna Tunali. These isolates were used in this study. Fungal isolates were cultivated at 26 ± 2 °C for 7 days. Experimental sets were grown on potato dextrose agar (PDA) medium supplemented with camphor (Sigma-Aldrich, St. Louis, MO, USA) with different concentrations (0.2, 0.4, 0.8, 1, 2, and 4 µg/µL). Subsequently, 0.25 cm^2^ agar plugs were used for in vitro growth. MIC and IC_50_ values were determined by using the agar dilution technique via measuring the radial growth [21].

### 4.2. Total RNA Extraction, cDNA Synthesis, and Gene Expression Assays

Total RNAs were extracted from the 7-day-old cultures using Tri-Reagent (Sigma-Aldrich, St. Louis, MO, USA). Fresh mycelium (50 mg) was homogenized with 0.8 mL Tri-Reagent according to the manufacturer’s recommendations. Total RNA molecules were analyzed with a spectrophotometer (Thermo Fisher Scientific, Massachusetts-USA) and agarose gel electrophoresis of 0.8% agarose gel.

The mRNA molecules were converted to cDNAs using a commercial kit (Takara, Shiga, Japan). The manufacturer’s protocol was followed. RNA (4 µg) was used as starting material in cDNA. The synthesized cDNA molecules were diluted according to four logarithmic phases.

The changes in gene expression due to camphor treatment were investigated by qPCR analysis. The endogenous gene was *β-tubulin*. Fold changes in *mgv1*, *stuA*, *CAT*, *POD*, *hog1*, *mst20*, and *tri5* gene expressions (which are closely related to essential cell processes in the fungal life cycle) were determined by 2^-ΔΔCT^ formula [31] (Table 4). Primer molecules were designed using “Primer3” software in the current study. A LightCycler 480 II (Roche, Basel, Swiss) system accompanied with Sybr Green I fluorescent dye was used in the qPCR assays. The reaction mix included 1× Sybr Green mix, 0.5 pmol forward/reverse primer, and an amount of cDNA equivalent to 2 µg RNA. Cycling conditions were 95 °C for 15 s, 57 °C for 15 s, and 72 °C for 20 s with 45 cycles. The pre-denaturation step at 95 °C for 2 min and a cooling step at 40 °C for 30 s were also carried out. Experiments were repeated at least three times.

### 4.3. Fluorescence Microscopy Assays

The apoptotic and oxidative stress effects of camphor on *F. graminearum* and *F. culmorum* were evaluated with AO/EB and DCF-DA staining by using fluorescence microscope. In AO/EB dual staining, mycelia from 7 days were harvested by centrifuging at 14,000 rpm for 3 min. The cells were washed twice with 1× phosphate buffered saline (PBS). Then, 5 µL AO/EB (60µg mL^−1^/100µg mL^−1^) mixture was added onto cells and they were incubated at 25 °C for 5 min. The cells were washed and treated with 1× PBS to remove excess AO/EB dye. Dual staining was screened by Texas RED and (green fluorescent protein (GFP) filters (Carl Zeiss, Oberkochen, Germany). In DCF-DA staining, DCF-DA was added to the cell suspension and incubated for 30 min at 25 °C. Then, the cells were washed twice with 1× PBS to eliminate excess DCF-DA and ROS activities were detected by fluorescein isothiocyanate (FITC) filter.

### 4.4. Antioxidant Activity and TLC

Total protein extraction from 0.5 gram of fresh mycelia from 7-day-old cultures was carried out by following the published protocol of Harris and Angal [32]. Protein concentrations were determined according to the protocol of Bradford [33]. The catalase (CAT) and peroxidase (POD) activities were obtained by spectrophotometric analysis at 240 nm and 470 nm, respectively. Antioxidant enzyme activities were calculated according to the normalization of the control and H_2_O_2_-treated series [34,35].

TLC was used to determine DON mycotoxin [36]. First, 10 mL of fungal cultures were filtrated by double gauze. Then, 1/5 volume of ethyl acetate was added for filtration. After centrifugation at 5000× *g* and 4 °C for 5 min, ethyl acetate was removed. Samples were dissolved with acetonitrile. In TLC, silica gel 60 F_254_ (Merck, Darmstadt, Germany), ethyl acetate:toluene (1:1), and ethanol:aluminium chloride (99:1) were used as the adsorbent, solvent, and reactive, respectively. The mycotoxin samples were co-spotted together with trichothecene standard (Sigma-Aldrich, St. Louis, MO, USA) on silica gels, and then run for 45–60 min. Silica gels were sprayed with reactive, and the Rf value was calculated via visualization at 365 nm UV light.

### 4.5. CRED-RA Analysis

gDNA was isolated from the 7-day-old cultures. Then, 100 mg mycelia were homogenized with liquid nitrogen and a sterile pestle and mortar. Next, 500 µL lysis buffer (100 mM Tris HCl, 100 mM EDTA, 1 M NaCl, 1% SDS, and 1/500: *v*/*v*: β-mercaptaethanol) was added and the homogenization was completed by grinding the samples. The sodium dodecyl sulphate-based protocol was then followed [37,38]. Qualitative and quantitative analyses of gDNA were performed via 1% agarose gel electrophoresis and a spectrophotometer (Thermo).

The RAPD method was used for the determination of the genomic template stability of *F. graminearum* and *F. culmorum* after camphor treatment. The CRED-RA method was used in the epigenetic profiling of camphor-treated and non-treated *F. graminearum* and *F. culmorum*. A routine RAPD protocol [39] was used in GTS assays of *MspI*-digested, *HapII*-digested, and non-digested control experimental sets. In total, 15 RAPD primers were used in this study (Table 2). gDNA digestion was carried out in a reaction volume of 20 µL including: 1× digestion buffer, 20 U digestion enzyme (Takara, Shiga-Japan), and 250 ng gDNA. The incubation was carried out at 37 °C for 1 h and 65 °C for 5 min. The digested and non-digested samples were used in CRED-RA assays. The amplicons were electrophoresed on 1.7% agarose gels and photographed via UV light. The RAPD band sizes were evaluated using a commercial 100 bp DNA size marker (Vivantis, Jaya, Malaysia). GTS percentage and the polymorphisms percentage for methylation were calculated as reported previously [40]. Amplification products, obtained from RAPD and CRED-RA, were evaluated according to Nardemir et al. [40].

### 4.6. Statistical Analysis

The qPCRs were statistically analyzed by GraphPad Prism 5.0 (Dr. Harvey Motulsky-GraphPad Company, La Jolla, CA, USA.) software by using one-way analysis of variance (ANOVA) with Tukey’s post-hoc test. A confidence interval of 0.05 was used. Standard deviation was calculated via column statistics.

## 5. Conclusions

The results of this study showed that camphor could lead to alterations at different molecular levels including epigenetics, antioxidant, gene expression, and apoptosis processes in *F. graminearum* and *F. culmorum*. This compound could be used as a single compound or combined with different chemicals which have fungicidal effects against *F. graminearum* and *F. culmorum*. Trees, producing the moderate or high level of camphor, might be also planted in fields where head blight and crown rot epidemics are present. However, further analyses should be carried out via in planta assays including plant tissue culture tests and field treatments in order to investigate the fungicidal effects of this kind of essential oil. Moreover, knowledge about the effects of camphor on other phytopathogenic microorganisms should be increased.

## Figures and Tables

**Table 1:** qPCR values of this study.

Gene	2^−ΔΔCT^ for 9F	2^−ΔΔCT^ for H-11
*hog1* (DQ065608.1)	2.178 ± 0.09	3.965 ± 1.42
*mst20* (XM_011329927.1)	1.821 ± 0.29	2.318 ± 0.33
*mgv1* (AF492766.1)	1.460 ± 0.39	1.826 ± 0.28
*stuA* (HG970332.2)	1.297 ± 0.14	6.359 ± 1.01
*CAT* (XM_011328072.1)	2.512 ± 0.22	1.996 ± 0.18
*POD* (XM_011329011.1)	1.241 ± 0.16	4.280 ± 0.94
*tri5* (AY130290.1)	0.449 ± 0.07	0.349 ± 0.02

**Table 2:** Random amplified polymorphic DNA (RAPD) primers used in this study and total band numbers obtained in RAPD analysis.

Primer	Sequence (5´–3´)	% GC	Total Band No.
OPA-01	CAGGCCCTTC	70%	13
OPA-03	AGTCAGCCAC	60%	9
OPA-04	AATCGGGCTG	60%	-
OPA-05	AGGGGTCTTG	60%	-
OPA-06	GGTCCCTGAC	70%	-
OPA-07	GAAACGGGTG	60%	9
OPA-08	GTGACGTAGG	60%	7
OPB-06	TGCTCTGCCC	70%	8
OPB-07	GGTGACGCAG	70%	5
OPB-09	TGGGGGACTC	70%	6
OPB-10	CTGCTGGGAC	70%	12
OPB-13	TTCCCCCGCT	70%	8
OPB-14	TCCGCTCTGG	70%	9
OPC-5	GATGACCGCC	70%	-
OPC-4	CCGCATCTAC	60%	-

**Table 3:** Band profiles obtained from coupled restriction enzyme digestion-random amplification (CRED-RA) analysis.

Primer	9F Control		9F Experiment Total Band Number		9F Experiment Total Polymorphic Band Number		9F Polymorphism (%)		H-11 Control		H-11 Experiment Total Band Number		H-11 Experiment Total Polymorphic Band Number		H-11 Polymorphism (%)	
			IC_50_		IC_50_		IC_50_				IC_50_		IC_50_		IC_50_	
	*HapII*	*MspI*	*HapII*	*MspI*	*HapII*	*MspI*	*HapII*	*MspI*	*HapII*	*MspI*	*HapII*	*MspI*	*HapII*	*MspI*	*HapII*	*MspI*
OPA1	3	4	3	3	0	1	0	25	4	5	8	2	5	3	100	60
OPA3	5	7	5	6	0	1	0	14.28	5	5	5	5	0	0	0	0
OPA7	3	3	3	4	0	1	0	33.33	3	3	6	3	3	0	100	0
OPA8	3	5	2	2	1	3	33.33	60	2	4	2	3	0	1	0	25
OPB6	5	7	4	5	1	2	20	28.57	4	5	5	6	3	1	75	20
OPB7	3	4	3	3	0	1	0	25	4	4	4	4	2	2	50	50
OPB9	3	3	3	3	0	0	0	0	4	4	4	4	2	2	50	50
OPB10	6	7	5	6	1	1	16.67	14.28	5	5	5	6	4	3	80	60
OPB13	5	5	5	5	0	0	0	0	5	5	5	4	0	1	0	20
OPB14	3	2	1	2	2	0	66.67	0	2	3	4	3	4	4	100	100
Mean	3.9	4.7	3.4	3.9	0.5	1	2.857143	18.33333	3.8	4.3	4.8	4	2.3	1.7	55.5	38.5

**Table 4:** Primers used in this study.

Primer	Primer Sequence (5′-3′)	Size (bp)
betaF/betaR	agggtcattacaccgagggt/gtaccaccaccaagagagtgg	121
MgvRTF/MgvRTR	aggttcaacgattccgacag/gaccattaccctgaggcaga	100
StuARTF/StuARTR	gcccctactggatacgatca/ttgccttctagggacattgg	100
Hog1F/Hog1R	cctggcaaaaatacgacgtt/tgatggagaattggttgacg	117
PodrtF/PodrtR	tggatcaaggacattggtga/gttggtagcatcctgctggt	117
tri5fullF/tri5fullR	atggagaactttcccaccgagtatt/agtccatagtgctacggataaggttcaa	469
CatrtF/CatrtR	aattccacgttcgtttcgtc/ccatactaggctcgctttgc	130
Mst20rtF/Mst20rtR	cctgaaaaggaacgacgaga/gccagcatgatggaatttct	117
